# Mapping evidence of the concomitant management of schistosomiasis by traditional health practitioners and health care professionals in communities with high prevalent infections: a systematic scoping review protocol

**DOI:** 10.1186/s13643-019-1088-3

**Published:** 2019-07-18

**Authors:** Gloire-Aimé Aganze Mushebenge, Tivani Mashamba-Thompson, Manimbulu Nlooto

**Affiliations:** 1 Discipline of Pharmaceutical Sciences, School of Health Sciences, University of KwaZulu-Natal, 6th Floor, E Block Building, Westville Campus, P B X54001, Durban, 4000 South Africa; 2 Discipline of Public Health Medicine, School of Nursing and Public Health, University of KwaZulu-Natal, 2nd Floor, George Campbell Building, Science Drive, Howard College Campus, Durban, 4001 South Africa

**Keywords:** Schistosomiasis, Neglected tropical diseases, Collaboration, Investigation, Traditional health practitioners, Health care professionals, Access, Low- and middle-income countries

## Abstract

**Background:**

Schistosomiasis is one of the most prevalent parasitic diseases in low- and middle-income countries (LMICs), being regarded as a neglected tropical disease in sub-Saharan Africa. Praziquantel is the conventional treatment recommended for schistosomiasis in mainstream healthcare systems. In many poor settings, while many people reportedly use both traditional medicine and public sector mainstream healthcare systems, little is known if those infected with schistosomiasis use both African traditional and prescribed antischistosomal medicines. This review aims to map evidence of the concomitant management of schistosomiasis by traditional health practitioners (THPs) and health care professionals (HCPs) in communities with a high prevalence schistosomiasis infection in LMICs.

**Methods/design:**

Guided by Arksey and O’Malley scoping review framework and Preferred Reporting Items for Systematic Reviews and Meta-Analyses (PRISMA), we will map the evidence from relevant studies dating from 2007 to 2019 published in LMICs. An electronic keyword search of the following databases will be conducted: PubMed, Cochrane Library, the Cumulative Index to Nursing and Allied Health Literature (CINAHL), and MEDLINE via EBSCOhost, Google Scholar, and WILEY online Library. Peer-reviewed articles, gray literature sources, and reference lists will be included to identify eligible studies. Following title screening, two reviewers will independently screen the abstracts and full texts. Any study that focuses on managing schistosomiasis will be included. The data will be analyzed using thematic analysis with the help of NVIVO software version 12, with the Mixed Method Appraisal Tool (MMAT) being used to assess the quality of the included studies.

**Discussion:**

This review will map the evidence in the literature of the concomitant management of schistosomiasis by THPs and HCPs in communities with a high prevalent infection in LMICs. The review findings will be important for policy makers across the healthcare continuum and be used to inform stakeholders’ consensus process to explore the development of a generic set of patient-centered quality indicators that are applicable to multiple care settings. It will also identify research gaps in schistosomiasis management in LMICs and provide direction for future research. The results will be disseminated through a peer-reviewed publication and presented in relevant conferences.

**Systematic review registration:**

PROSPERO CRD42017078198

**Electronic supplementary material:**

The online version of this article (10.1186/s13643-019-1088-3) contains supplementary material, which is available to authorized users.

## Background

Infectious diseases are both a major public health concern and a socioeconomic problem in tropical regions within most low- and middle-income countries (LMICs), where neglected tropical diseases (NTDs), such as schistosomiasis, are cause for concern [1]. These NTDs often occur in people who are also infected with human immunodeficiency virus (HIV), malaria, or tuberculosis, making the problem even more serious, as co-infections are common [2]. Globally, more than 250 million people are infected with schistosomiasis, with approximately 700 million at risk of infection [3]. Schistosomiasis is a parasitic disease caused by schistosomes, worms that are found in tropical and sub-tropical fresh waters [4]. An estimated 206.4 million people needed preventive treatment for schistosomiasis in Africa in 2016, of whom approximately 89 million (43%) were reportedly treated [5]. More than five million people in South Africa, mainly in rural areas, required treatment in 2014 for urogenital schistosomiasis [6].

Praziquantel, the recommended treatment against all forms of schistosomiasis [7], is inexpensive and regarded as effective and safe, although re-infection may occur after treatment. The risk of developing severe disease is reduced when treatment is initiated and repeated in childhood [8]. Many countries, such as Mozambique, Zambia, Nigeria, and Uganda, have implemented mass treatment campaigns since the 1980s with generic medicines, including praziquantel, with some having attained countrywide coverage the last few years [9–11]. The cost of praziquantel in South Africa is 50 times higher than the World Health Organization (WHO) standard treatment that is in use in the rest of Africa, making it costly to provide mass treatment, which cannot be implemented [12]. None of the companies producing generic praziquantel in other countries have the right to sell them in South Africa, due to the time-consuming, expensive, “scientifically unnecessary,” and elaborate registration process [13]. However, the cost of praziquantel in South Africa, despite having been reduced, remains prohibitive for many control programs in schistosomiasis endemic areas [14].

Alternatively, traditional medicine has demonstrated its contribution to managing schistosomiasis [15]. In an ethnopharmacological survey conducted in Niger and Mali, 55 plant species were reported to be used for treating schistosomiasis either alone or in combination, of which *Zea mays* with *Glossonema boveanum* were specific for intestinal schistosomiasis, while *Cissus quadrangularis* and *Stylosanthes erecta* were reported for the first time in Mali to be used against urogenital schistosomiasis [16]. A range of medicinal plants with anti-schistosomiasis properties have been widely used by traditional healers of different tribes in South Africa, although their effectiveness has not been scientifically evaluated [14]. A study conducted on mice in Zimbabwe showed no significant difference between an herbal preparation (Schitozim) and praziquantel in managing schistosomiasis. However, the authors warranted further investigation to determine the toxic levels and effective doses of Schitozim in humans [17].

Due to the unavailability of conventional therapy, or the expensive cost of praziquantel and other antischistosomal medicines, people in rural areas may not have access to modern treatment for schistosomiasis, which results in them using traditional medicine [18, 19]. Collaboration between the two medical traditions can provide appropriate care for diseases management, for example, through mutual referral [20].

This review aims to map the evidence of the concomitant management of schistosomiasis by traditional health practitioners and health care professionals in communities with a high prevalence of schistosomiasis infection in LMICs. Thus, the purpose of this review is to provide evidence to enable the implementation of policies and guidelines to manage schistosomiasis with traditional medicine contributing to primary healthcare.

## Methods/design

### Scoping review framework

This review will be based on the framework originally proposed by Arksey and O’Malley [21] and further improved by Levac et al. [22], which are presented in Table 1 [21–23]. There are six steps involved in the framework, although the last step about consulting experts/stakeholders will not be conducted for this review due to funding constraints. Consulting additional sources of information, perspectives, meaning, and applicability will be covered with gray literature. This review follows the six steps outlined by the Arksey and O’Malley framework, but will incorporate enhancement suggested by the later authors.

**Table 1:** Scoping review framework for this review [21–23]

Arksey And O’Malley framework	Enhancements proposed by Levac, Colquhoun, and O’Brien
1. Identify the research question	Clarify and link the purpose and research question
2. Identify relevant studies	Balance the feasibility with breadth and comprehensiveness of the scoping process
3. Select the study	Use an iterative team approach to select studies and extract data
4. Chart the data	Incorporate a numerical summary and qualitative thematic analysis
5. Collate, summarize, and report the results	Identify the implications of the study findings for policy, practice, or research
6. Consult experts/stakeholders (optional)	Provide opportunities for consumer and stakeholder involvement to suggest additional references and provide insights beyond those in the literature

#### Identify the research questions

The following research questions are formulated to guide the review in meeting its aims and objectives. The general research question is: “What is the evidence about the concomitant management of schistosomiasis by THPs and HCPs in communities with a high prevalence schistosomiasis infection?” The specific research questions are as follows:

1. What is the evidence of the healthcare seeking behavior among individuals in communities with high prevalent infections of schistosomiasis?
2. What is the evidence of the use of traditional, complementary, and alternative medicine for managing schistosomiasis?
3. Is there a bidirectional referral of patients between THPs and HCPs for managing schistosomiasis?

##### Eligibility of research questions

The Population, Intervention, Comparison and Outcomes (PICO) for the research questions has been used to break down the clinical questions into searchable keywords (See Table 2) [24].

**Table 2:** PICO framework

Framework	Evidence-based practice
P: Population	THPs and HCPs, aged 18 years old and above.
I: Intervention	Traditional remedies for schistosomiasis management, praziquantel, and LMICs.
C: Comparison	Schistosomiasis management by THPs and HCPs.
O: Outcomes	Access to treatment and improvement of the management of schistosomiasis in communities with high prevalent infection.

#### Identify relevant studies

Relevant literature will be searched from the following databases: PubMed, EBSCOhost (the Cumulative Index to Nursing and Allied Health Literature (CINAHL), MEDLINE, Google Scholar, Cochrane Library, WILEY online Library, and grey literature. Reference lists of included studies will also be searched. The keywords search will include the following: schistosomiasis; neglected tropical diseases; collaboration; investigation, traditional health practitioners; health care professionals; access; low- and middle-income countries, schistosomiasis, or collaboration or traditional medicine or neglected tropical diseases (NTDs) or natural products or leads. The Boolean search terms (AND and OR) and MeSH terms (“therapy”, “therapeutics”, “schistosomiasis”, “health personnel”, “residence characteristics”, “infection”, …) will be included in the search. Gray literature will be identified through website source links in references. Peer review studies and gray literature reporting on the evidence of managing schistosomiasis by THPs and HCPs in LMICs published between 2007 and 2019 will be included. Authors of primary studies or reviews will be contacted for further information or to access missing studies where relevant. If the authors do not respond, then their publications will be excluded.

#### Select the study

##### Inclusion criteria

The following criteria provide a guide to clearly understand what is proposed by the reviewers and, more importantly, a guide for the reviewers themselves upon which to base decisions about the sources to be included in this review [25]. As explained above, regarding the review types, there must be clear congruency between the title, objectives, question/s, and inclusion criteria of a scoping review. They are as follows:

1. Evidence of the population of interest in this review (THPs and HCPs)
2. Evidence of the intervention (schistosomiasis treatment and co-morbidities)
3. Evidence of the comparison of treatment by THPs and HCPs
4. Evidence on the outcomes of managing schistosomiasis
5. Articles published in English and French will be included.

##### Exclusion criteria

1. Articles published before 2007.
2. Articles not addressing the treatment of schistosomiasis and comorbidities.
3. Articles not addressing integration and collaboration between traditional medicine and conventional medicine.
4. Studies that are not reporting evidence from LMICs

##### Search strategy

The search strategy for this review aims to be comprehensive to identify both published and unpublished (grey literature) primary studies and reviews [25, 26]. A pilot search was conducted with the database results provided in Additional file 1. A three-step search strategy will be utilized. The first step is an initial limited search of the electronic databases. This initial search will be monitored, exported on EndNote X9 reference manager for abstract and full article screening. The duplicated article will be deleted. A second search using all identified keywords and index terms will then be undertaken across all included databases. Thirdly, the reference list of all identified reports and articles will be searched for additional studies [25]. For abstract and full article screening, the EndNote library will be shared with a second reviewer. Any discrepancies in the results of the abstract screening will be resolved through a discussion until consensus is reached. A third screener will help resolving discrepancies in full article screening results [26].

**Fig. 1:**
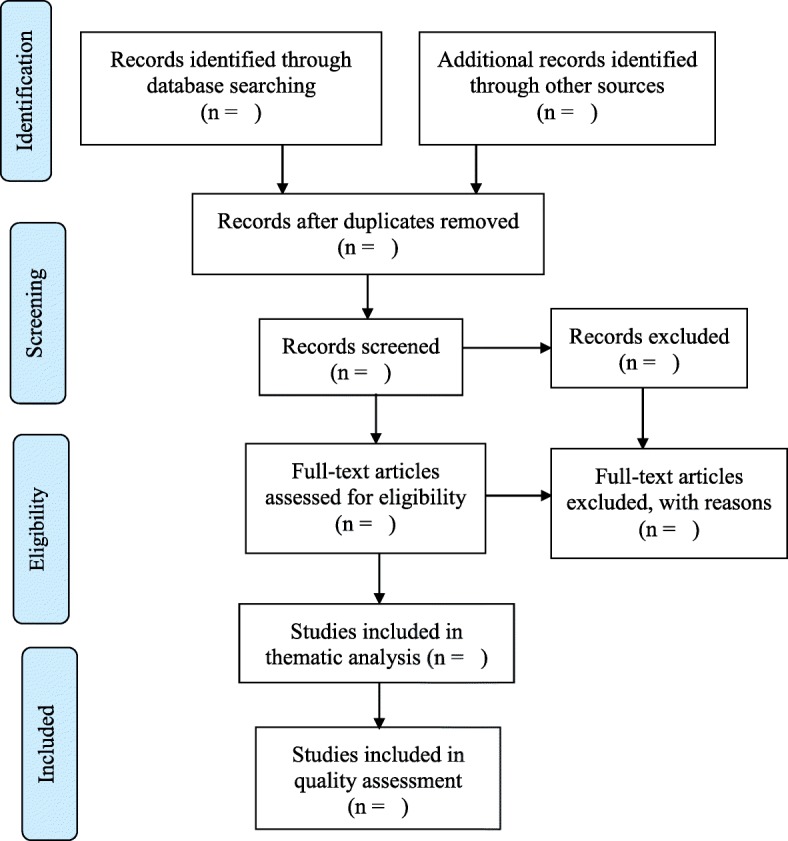
The PRISMA flow diagram for the scoping review screening process [25]

Publications duplicated in the research results will be treated as a single study for the review. To maintain transparency in the review selection process, a PRISMA Flow Diagram will be followed in each stage of the selection process. In addition, a list of the studies excluded during the full-text review will be documented as an Additional file 2, with brief reasons for their exclusion [26]. A PRISMA Flow Diagram will be used to report the screening results (see Fig. 1). EndNote will help to manage the search results including downloading all results, removing duplicate records, and screening for potentially relevant studies.

##### Quality appraisal

For the quality appraisal of the included studies, we will use a Mixed Methods Appraisal Tool (MMAT), version 2018 [27]. The MMAT will allow us to assess the appropriateness and quality of the research. Studies can be scored following specific criterion using a certain score to describe them from 50% and above.

#### Chart the data

We will conduct data extraction to enable a logical and descriptive summary of the search results (Additional file 1) of our systematic scoping review [25, 26]. A table of characteristics for included studies will be developed at this stage to record the key information of the source, such as author, reference, and results or findings, relevant to the review question/s (Additional file 3). This may be further refined at the review stage and the sample extraction form updated accordingly. From the key information chart, data in the selected articles will be in data extraction form and synthesized into different themes for interpretation to identify key findings.

#### Collate, summarize, and report the results

The results will be presented in a data extraction form or table and will be further refined towards the end of the review when the authors will have the greatest awareness of the contents of their included studies to manage schistosomiasis. The results will be mapped in existing thematic framework, which will consist of the included papers in a diagrammatic or tabular form, and/or in a descriptive format that aligns with the objective/s and scope of the review related to outcomes. The PICO elements for inclusion criteria will be useful to guide how the data will be mapped most appropriately.

A narrative summary will accompany the tabulated and/or charted results and should describe how they relate to the review objective and question/s according to the management of schistosomiasis and its co-morbidities by THPs and HCPs. The synthesis of important findings across the included studies will be classified by identifying prominent themes under main conceptual categories, such as: “intervention type”, “review population” (and sample size, if it is the case), “duration of intervention”, “aims”, “methodology adopted”, “key findings” (evidence established), and “gaps in the research”. For each category reported, a clear explanation will be provided.

##### Synthesis

Throughout this research, we will examine the above-mentioned themes and critically identify their link to the research question. Reviewers will analyze the significance of the findings according to the aim of this research and their implications for future studies, practice, and policy.

## Discussion

This review is part of larger studies evaluating the management of schistosomiasis by THPs and HCPs in South Africa. There is little evidence to support the current South African policy to manage schistosomiasis. This systematic scoping review aims to build on the work of the existing Cochrane study [20] by further describing the participant inclusion criteria and utilizing a wider range of evidence on this topic. A key strength is that it can provide a rigorous and transparent method for mapping areas of research according to the treatment of schistosomiasis with comorbidities. We wish to be able to illustrate the field of interest, that being the management of schistosomiasis, in terms of the volume, nature, and characteristics of the primary research.

Articles not addressing integration and collaboration between traditional and modern medicine will not be part of this research, as it intends finding a way to manage schistosomiasis via bidirectional referral of patients between THPs and HCPs. Due to the prevalence of schistosomiasis, a collaboration of THPs and HCPs could help to manage the infection, as traditional medicine is available to most people in LMICs, with not less than 80% of people worldwide depending on it [28].

It is anticipated that this review will identify gaps in the current literature on this topic and provide direction for future research in this area of this review. The summary and dissemination of these research findings may be of interest for policy makers and stakeholders (practitioners and consumers) who are involved in the NTDs management, especially those involved in schistosomiasis management using either the mainstream healthcare systems or African traditional medicine.

## Additional files

Additional file 1: Results of pilot database search. (DOCX 14 kb)

Additional file 2: PRISMA-P (Preferred Reporting Items for Systematic review and Meta-Analysis Protocols) 2015 checklist: recommended items to address in a systematic review protocol. (DOCX 22 kb)

Additional file 3: Sample extraction form. (DOCX 15 kb)

## Data Availability

All data generated or analyzed during this review will be included in the published scoping review

## Abbreviations

HCPs: Health care professionals
HIV/AIDS: Human Immunodeficiency Virus/Acquis immunodeficiency deficiency Syndrome
LMICs: Low- and middle-income countries
MMAT: Mixed Methods Appraisal Tool
NTDs: Neglected tropical diseases
PICO: Population Intervention Comparison Outcomes
THPs: Traditional healthcare practitioners
TM: Traditional medicine
