# Supplementary material for: Mapping evidence of the concomitant management of schistosomiasis by traditional health practitioners and health care professionals in communities with high prevalent infections: a systematic scoping review protocol
Source: Syst Rev. 2019 Jul 18;8:175. doi: 10.1186/s13643-019-1088-3 (PMC6637472; doi:10.1186/s13643-019-1088-3)
Supplement: Supplementary file 1 — Results of pilot database search. (DOCX 14 kb) [file 13643_2019_1088_MOESM1_ESM.docx]

***Table S1: Results of pilot database search***

| Keyword Search | Date of search | Search Engine used | Number of publications retrieved (Results) |
| --- | --- | --- | --- |
| Mapping[All Fields] AND evidence[All Fields] AND concomitant[All Fields] AND ("therapy"[Subheading] OR "therapy"[All Fields] OR "treatment"[All Fields] OR "therapeutics"[MeSH Terms] OR "therapeutics"[All Fields]) AND ("schistosomiasis"[MeSH Terms] OR "schistosomiasis"[All Fields]) AND Traditional[All Fields] AND ("health"[MeSH Terms] OR "health"[All Fields]) AND Practitioners[All Fields] AND ("health personnel"[MeSH Terms] OR ("health"[All Fields] AND "personnel"[All Fields]) OR "health personnel"[All Fields] OR ("health"[All Fields] AND "care"[All Fields] AND "professionals"[All Fields]) OR "health care professionals"[All Fields]) AND ("residence characteristics"[MeSH Terms] OR ("residence"[All Fields] AND "characteristics"[All Fields]) OR "residence characteristics"[All Fields] OR "communities"[All Fields]) AND high[All Fields] AND prevalent[All Fields] AND ("infection"[MeSH Terms] OR "infection"[All Fields] OR "infections"[All Fields]) AND low[All Fields] AND middle[All Fields] AND ("income"[MeSH Terms] OR "income"[All Fields]) AND countries[All Fields] | September 18^th^ 2017 | Google Scholar, PUBMED | 8370 |
