# Supplementary material for: Mapping evidence of the concomitant management of schistosomiasis by traditional health practitioners and health care professionals in communities with high prevalent infections: a systematic scoping review protocol
Source: Syst Rev. 2019 Jul 18;8:175. doi: 10.1186/s13643-019-1088-3 (PMC6637472; doi:10.1186/s13643-019-1088-3)
Supplement: Supplementary file 3 — Sample extraction form. (DOCX 15 kb) [file 13643_2019_1088_MOESM3_ESM.docx]

***Table S3: Sample extraction form***

| Key information chart |
| --- |
| 1. *Title* |
| 1. *Author(s)* |
| *c. Year of publication* |
| *d. Origin/country of origin (where the study was published or conducted)* |
| *e. Aim (s) /purpose(s)* |
| *f. Summary of the study results* |
| *g. Study population and sample size (if applicable)* |
| *h. Methodology/methods (recruitment and sampling)* |
| *i. Study design* |
| *j. Data collection method* |
| *k. Data analysis* |
| *l. Intervention type, comparator and details of these*  *(e.g. duration of the intervention) (if applicable)* |
| *m. Duration of the intervention (if applicable)* |
| *n. Outcomes and details of these (e.g. how measures) (if applicable)* |
| *o. Relevant Key findings that relate to the scoping review question/s.* |
| *p. Conclusions* |
| *q. Comments* |
